# Neurotrophic factors in bipolar disorders patients with manic episode

**DOI:** 10.3906/sag-1907-70

**Published:** 2020-06-23

**Authors:** Özgür Korhan TUNÇEL, Gökhan SARISOY, Eda ÇETİN, Ebru Kaynar TUNÇEL, Birşen BİLGİCİ, Arzu KARAUSTAOĞLU

**Affiliations:** 1 Medical Biochemistry Department, Faculty of Medicine, Ondokuz Mayıs University, Samsun Turkey; 2 Psychiatry Department, Faculty of Medicine, Ondokuz Mayıs University, Samsun Turkey; 3 PublicHealth Center, Samsun Turkey

**Keywords:** Bipolar disorder, neurotrophic factors, IGF-1, GDNF, neuritin 1

## Abstract

**Background/aim:**

Neurotrophins are one of the most important molecule groups affecting cerebral neuroplasticity. The amount of evidence about the role of changes in neuroplasticity in the pathophysiology of bipolar disease is growing.

**Materials and methods:**

We measured serum levels of brain-derived neurotrophic factor (BDNF), nerve growth factor (NGF), neurotrophin-3 (NT-3), glial cell-line derived neurotrophic factor (GDNF), vascular endothelial growth factor (VEGF), insulin-like growth factor-1 (IGF-1), fibroblast growth factor (FGF)-2, neuritin 1 (Nrn 1) in bipolar 1 manic episode patients (n = 45) and healthy control group.

**Results:**

When controlled for age, BMI and cortisol, it was found that the serum levels of BDNF, NGF, NT-3, VEGF and FGF-2 of bipolar manic episode patients were not statistically different compared to those of the control group. GDNF level and Nrn 1 levels were significantly lower (P = 0.003 and P = 0.025 respectively) while IGF-1 levels were significantly higher than the control group (P = 0.0001). ROC analysis was performed and the area under the the curve was calculated as 0.737, 0.766 for GDNF, IGF-1 respectively.

**Conclusion:**

The changes in the levels of GDNF, IGF-1 and Nrn 1 might be involved in pathopysiology of bipolar disorder, and GDNF, IGF-1 may be considered as state markers in bipolar manic episode.

## 1. Introduction

Bipolar disorder is a multifactorial disease in which inflammation, oxidative stress and neurotropic factors play a role [1]. All these factors affect neuroplasticity and the impairment in neuroplasticity has a central role in the pathophysiology and treatment of bipolar disorder [2].

Neurotrophins are the principal regulators affecting neuroplasticity and these proteins are crucial in neural survival, growth and functioning [3]. BDNF, NGF and NT-3, the neurotropic factors, are synthesized as proneurotrophin and are converted into mature neurotrophines by cleavage. These proneurotrophines bind to Trk and P75NTR receptors and modify neural survival, growth and plasticity by activating signal pathways including Ras/ERK, PI3k/AKT and PLCϒ [4].The homeostasis disturbances in these neurotrophic factors may contribute to cerebral atrophy and progressive cognitive disorder observed in bipolar disorder [5,6,7].

GDNF is a member of transforming growth factor β super family and is synthesized in many parts of the brain [8]. It is not only related with the dopaminergic neurons in mesencephalon [9] but also with the growth of central and peripheral nervous system and protection of plasticity [10]. GDNF may have this effect by reducing the oxidative stress [11]. However, there are a limited number of studies on the relationship between bipolar disorder and the level of GDNF and, there is no consensus on the results of these studies [12].

VEGF, IGF-1 and FGF-2 are called angioneurins. They are growth factors with both neurotropic and neurovascular effects [13]. VEGF increases neuron proliferation in hippocampus [14] and, genetic variations of VEGF are associated with the morphology of hippocampus [15]. Increased VEGF level may be an indicator of compensatory response to the restoration of neurogenesis in bipolar patients at manic episode [16]. IGF-1 is a growth factor most of which is synthesized out of the central nervous system, namely in liver, and affects synaptic plasticity by passing the blood-brain barrier. It achieves this effect via glutamatergic synaps [17]. It was observed that IGF Binding Protein-2 (IGFBP-2) level decreased in the brain in bipolar disorder [18]. However, increased peripheral IGF-1 levels in bipolar disorder may be related with the absence of negative feedback resulting from the reduced central effect of IGF-1 [19]. FGF-2 is produced in many parts of the brain and is important in neural survival and growth [20]. Reduced expression of this neurotropic factor and its receptors may be related with mental diseases [21]. There is only one study investigating the relationship between bipolar disease and FGF-2 and it suggests that FGF-2 levels increase in manic episode [13]. Yet, further studies on the subject are required. 

Neuritin is a protein which supports neuritogenesis, particularly in hippocampus and cortical cells, by affecting glycosylphosphatidylinositol [22]. Nrn 1 is aneuritin homolog and regulates dendritic and axonal branching [23]. It also increases synaptic maturation and neural migration [24]. It is proposed that reduced neuronal dendritic branching, hippocampus plasticity and limbic structure are related with the mood disorders. Therefore, changes in the expression of neuritin may be related with mood disorders. It was reported that application of chronic stress reduced the expression of neuritin in hippocampus and that the use of antidepressants reversed this process [25]. In another study conducted on schizophrenia patients, it was proposed that impairment in general intelligence was related with polymorphism in Nrn 1 gene [26]. In the light of this information, changes in the level of Nrn 1 may play a role in the etiopathogenesis of bipolar disorder. We have not found any study in literature on the relationship between bipolar disorder and neuritin 1.

It is proposed that abnormal cortisol metabolism is related with the disfunctioning of hypothalamo-hypophyseal axis in bipolar patients [27]. Studies conducted before 2014 were reviewed in a recent meta-analysis and it was found that the cortisol level increased slightly in bipolar patients during depressive and euthymic episodes [28]. Increased cortisol levels may play a role by decreasing the levels of neurotrophic factors [29]. In addition, another factor which may have an effect on neurotropic factor is obesity. [30]. Neurotropic factors seem to be effective in body weight control and it argued that there is a significant reduction of these factors in obese individuals [31]. Another factor is ageing and metabolic changes in brain during the ageing process may be accompanied by a decrease in the expression of neurotropoic factors [32]. Thus, studies on this subject need to consider the relationship between the age, BMI, cortisol levels, and neurotrophic levels of the patients. 

In the light of the abovementioned information, there may be a relation between bipolar disorder and neurotropic factors (BDNF, NGF, NT-3, GDNF, VEGF, IGF-1, FGF-2 and Nrn 1). However, the research on this subject is limited and is far from revealing the relation between neurotropic factors and bipolar disorder. We aimed to reveal whether there was a change in neurotropic factors during the manic episode of type 1 bipolar patients by keeping the effects of potential confounding factors such as age, BMI and cortisol level. We also analyzed the relationship between these parameters and the sociademographic and clinical features of the disorder.

## 2. Material and methods

### 2.1. Subjects

This study was conducted on bipolar disorder Type 1 manic episode patients of 18 to 65 years old who applied to Ondokuz Mayıs University, Faculty of Medicine, Department of Psychiatry (n = 45) and a control group (n = 45). All subjects were evaluated by at least 2 psychiatrists. Diagnosis of bipolar disorder manic episode was made using DSM-IV (The diagnostic and statistical manual of mental disorders, 4th edition). The mania severity was measured using YMRS (young mania rating scale) [33]. Comorbidity was evaluated for all subjects using SCID-I (structured clinical interview for DSM-IV Axis I disorders) [34] and SCID-II (structured clinical interview for DSM-III-R personality disorder) [35]. Subjects with a potential of psychiatric comorbidity were excluded from the study. The control group matched with patient groups regarding age and sex and had never had a psychiatric disease. All subjects had a body index of ≤ 25 kg/m2 and were nonsmokers. In addition, none of the subjects had drug addiction, pregnancy, breastfeeding or chronic disease (infection, inflammatory disease, diabetes, hypertension, cancer and etc.). This study was approved by Ondokuz Mayıs University, Ethical Committee of Clinical Research (No: 2014/755 Issue B.30.2.ODM.0.20.08/162). All procedures were organized in accordance with World Health Organization’s Helsinki Declaration. All subjects were informed and written consent was received. 

### 2.2. Collection of blood

5 mL of blood was taken from each participant into the biochemistry tubes after 12 h fasting at 8:00–10:00 AM. The blood collected was centrifugated (Jouan C4i Cat no: 11177560) at 3000 g, 5 min, at 4 oC. The separated serum was stored at –80 oC (NUAIRE Ultra-Low Freezer Model no: Nu-6420E) until biochemical analyzes.

### 2.3. Biochemical measurements 

BDNF levels were measured using Human BDNF Elisa Kit (Boster, California, USA. Cat No: EK0307 Lot No: 361144504). The sensitivity was <2 pg/mL, intraassay and interassay CV were 4.2% and 7.5% respectively. NGF levels were measured using Human NGF/NGF-β Elisa Kit (Boster, California, USA. Cat No: EK0469 Lot No: 1881116427). The sensitivity was <1 pg/mL, intraassay and interassay CV were 7.2% and 8.1% respectively. NT-3 levels were measured using Human Neurotrophin-3 Elisa Kit (Boster, California, USA. Cat No: EK0472 Lot No: 19110128427). The sensitivity was <2 pg/mL, intraassay and interassay CV were 4.9% and 6.1% respectively. GDNF levels were measured using Human GDNF Elisa Kit (Boster, California, USA. Cat No: EK0362 Lot No: 851034427). The sensitivity was <4 pg/mL, intraassay and interassay CV were 5.7% and 8.2% respectively. VEGF levels were measured using Human VEGF Elisa Kit (Boster, California, USA. Cat No: EK0539 Lot No: 2531133427). The sensitivity was < 1 pg/mL, intraassay and interassay CV were 4.2% and 7.5% respectively. IGF-1 levels were measured using IGF-1-EASIA Kit (DIAsource, Louvain-la-Neuve, Belgium, Katalog No: KAP1581, Lot No: 151101). The detection limit was 7.8 ng/mL, intraassay and interassay were CV 6.1% and 12.9% respectively. FGF-2 levels were measured using Human (FGF2) ELISA Kit (Sunred, Shanghai, China, Katalog No: 201-12-0056, Lot No: 201505). The detection limit was 4.458 ng/mL, intraassay and interassay CV were <10% and <12% respectively. Nrn 1 levels were measured using Human Neuritin (NRN1) ELISA Kit (CUSABIO, Wuhan, China, Katalog No: CSB-EL016088HU, Lot No: K11162142). The detection limit was 7.8 pg/mL, intraassay and interassay CV were <8% and <10% respectively. Cortisol levels were measured with chemiluminescence method using Roche Diagnostic E170 autoanalyzer. The detection limit was 1.5 nmol/L, intraassay and interassay CV were 2.4% and 2.8% respectively.

### 2.4. Statistical analysis 

SPSS for Windows 15.0. was used for statistical analysis. The normal distribution of the data was checked with Kolmogorov-Smirnov normal distribution test. The comparison of categorical data was analyzed with chi-square test while continuous parameters were compared with independent samples ttest and Mann-Whitney U test. To control the effect of potential confounding factors (age, BMI and cortisol level) on statistical difference, analysis of covariance (ANCOVA) was used. We conducted correlation analyses for biochemical parametres themselves. We also analyzed the correlation between biochemical parametres and, clinical and demographical features of the patients. Pearson and Spermann correlation method were used. To control the effect of the confounding factors on correlations, partial correlation analysis was applied. ROC analysis was conducted in order to evaluate diagnostic value of biochemical parametres for which there were statistical differences among the groups. A value of P < 0.05 was considered as significant. Results were given as mean ± standart deviation. 

## 3. Results

There was no statistical difference between the control group and the bipolar manic episode patients with respect to the age, sex and BMI (P > 0.05). There were differences between the groups with respect to the socioeconomic status (P < 0.05). Of all the patients, 3 (6.7%) patients had suffered 1, and 4 (8.9%) patients had suffered 2 attacks of hypomania. Two of the patients (4.4%) had had a mixed attack while 17 patients (37.8%) had had only one depression attack. Clinical and demographical features of the subjects are presented in Table 1. 

**Table 1:** Clinical and demographical features of the subjects.

	Control (n = 45)	Patient (n = 45)	P
Age, mean ± SD (years)	34.91 ± 10.76	34.91 ± 10.76	1.00a
Sex, n (%)			
Male	23 (51.1)	23 (51.1)	1.00b
Female	22 (48.9)	22 (48.9)	
Socioeconomical status, n (%)			
Low	3 (6.7	8 (17.78	0.014b
Middle	41 (91.1)	30 (66.67)	
High	2 (2.2)	7 (15.56)	
BMI (kg/m2), mean ± SD	21.95 ± 2.4	21.27 ± 2.12	0.177b
YMRS, mean ± SD		40.11 ± 8.12	
Number of episode, mean ± SD		5.22 ± 4.55	
Number of manikepisode,mean ± SD		4.00 ± 3.66	
First episode, n (%)			
Mania		13 (28.9)	
Depression		32 (71.1)	
Age of onset, mean ± SD (years)		25.00 ± 8.78	
Duration of disease, mean ± SD (years)		9.82 ± 6.86	
Suicide attempt n (%)		5 (11.1)	
Family history for psychiatric disorder n (%)		10 (22.2)	
Bipolar disorder		7 (15.6)	
Schizophrenia		1 (2.2)	
Depression		2 (4.4)	
Medicines used, n (%)			
Lithium carbonate		14 (31.1)	
Valproic acid		30 (66.7)	
Lamotrigine		1 (2.2)	
Antipsychotics,n (%)			
Non		1 (2.2)	
First generation		2 (4.4)	
Second generation		34 (75.6)	
First + Second generation		8 (17.8)	

a Independent samples t-test, bChi-square test.

In the first statistical analysis disregarding the confounding factors (age, BMI, cortisol), it was revealed that there was no difference between the groups regarding cortisol, BDNF, NGF and NT-3 levels (P = 0.154, P = 0.178, P = 0.205 and P = 0.155 respectively). However, there were statistical differences between the groups with regard to GDNF, VEGF, IGF-1, FGF-2 and Nrn 1 levels (P values were P = 0.001, P = 0.023, P = 0.000015, P = 0.013, P = 0.021 respectively). In the second statistical analysis, age, BMI and cortisol were taken under control to eliminate the effects of these parametres. The statistical analysis showed that statistical difference persisted in GDNF, IGF-1 and Nrn 1 levels (P = 0.003, P = 0.0001 and P = 0.025 respectively) while there were no statistical differences in VEGF and FGF-2 levels (P = 0.149 and P = 0.078 respectively). All data are presented in Table 2. 

**Table 2:** Biochemical parametres.

	Control (n = 45)	Patient (n = 45)	P	U,t,F value
BDNF (pg/mL)	6551.00 ± 1912.69	7182.32 ± 1638.35	0.178a	M-W U = 826
NGF(pg/mL)	36.29 ± 14.53	32.59 ± 12.20	0.205b	t = 1.277
NT-3 (pg/mL)	89.59 ± 60.87	110.68 ± 70.35	0.155b	t = –1.437
GDNF (pg/mL)	2785.31 ± 1255.91	1733.47 ± 1201.19	0.003c	F = 9.335
VEGF (pg/mL)	52.69 ± 24.12	62.25 ± 24.67	0.149c	F = 2.125
IGF-1 (ng/mL)	190.68 ± 56.58	279.30 ± 139.54	0.0001c	F = 15.930
FGF-2 (ng/mL)	837.68 ± 432.98	653.38 ± 404.78	0.078c	F = 3.176
Nrn 1 (pg/mL)	173.89 ± 114.38	121.19 ± 72.38	0.025c	F = 5.281
Cortisol	14.43 ± 5.24	12.92 ± 4.64	0.154b	t = 1.439

BDNF: Brain-derived neurotrophic factor, NGF: Nerve growth factor, NT-3: Neurotrophin-3, GDNF: Glial cell-line derived neurotrophic factor, VEGF: Vascular endothelial growth factor, IGF-1: Insulin-like growth factor-1, FGF-2: Fibroblast growth factor, Nrn 1: neuritin 1. aMann-Whitney U test, bIndependent samples t-test, cAnalysis of covariance (ANCOVA) controlling for age, BMI and cortisol.

When age, BMI and cortisol level were taken under control in the correlation analysis, there was a statistically significant correlation between only Nrn 1 and BDNF in the control group (r = 0.383, P < 0.021). In the patient group, there was no correlation neither among the biochemical parametres themselves nor the clinical and demographical features of the patients. 

**Figure 1:**
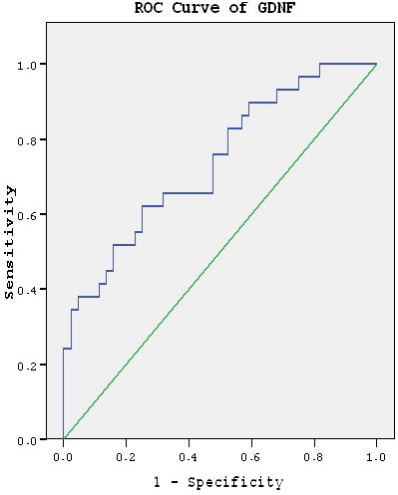
ROC curve for GDNF.

**Figure 2:**
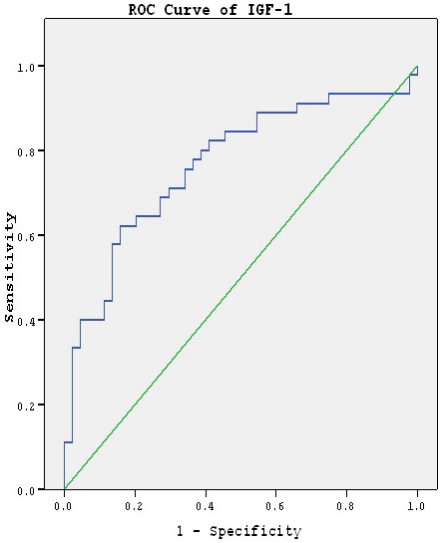
ROC curve for IGF-1.

ROC analysis was performed on the biochemical parametres in which the groups had statistical differences and the area under the the curve was calculated as 0.737, 0.766 for GDNF and IGF-1, respectively. (Figures 1 and 2, Table 3)

**Table 3:** ROC analysis values.

Test result variable(s)	Area	Std. error (a)	Asymptotic sig. (b)	Asymptotic 95% confidence interval	
				Upper bound	Lower bound
GDNF	0.737	0.059	0.001	0.621	0.854
IGF-1	0.766	0.051	0.000	0.666	0.867
Nrn 1	0.626	0.067	0.069	0.495	0.758

a: Under the nonparametric assumption, b: Null hypothesis: true area = 0.5.

## 4. Discussion

The evidence about structural abnormalities of the brain in bipolar disorder is increasing [36]. Neurotrophic factors are among the most important factors underlying these changes. Although the data collected on the role of neurotrophines in the pathophysiology of bipolar disorder has increased, they are conflicting and far from being sufficient [12]. Moreoever, it is argued that these neurotropic factors also change in other psychiatric diseases such as major depression and schizophrenia, which implies that the change in these factors may not be unique to bipolar disorder [37,38]. In order to reveal this point, we kept cortisol level, BMI and age under control statistically in the groups and we found that GDNF and Nrn 1 levels were significantly lower while IGF-1 levels were significantly higher in bipolar manic episode patients compared to the control group. On the other hand, there were no differences among the groups with respect to BDNF, NGF, NT-3, VEGF and FGF-2 levels.

BDNF is the most commonly studied neurotrophic factor in bipolar disorder. There are studies in which BDNF increased [39, 40], decreased [41] or remained unchanged [42]. However, the decrease in BDNF may particularly be observed in long-term bipolar patients [43]. Two postmortem studies also showed that BDNF decreased in brain tissue [6, 44]. In order to minimize the effects of confounding factors, we matched the patient group and the control group with respect to age and sex. In addition, patients with a body index of BMI >25 were excluded. We found no difference in BDNF levels. Possibly, the BDNF level could be normalized as a result of medication. Although there are studies on this subject, it is not possible to explain this result clearly because medication-free patients were not examined sufficiently [41, 45]. Another factor is the possiblity of reaching different results at different episodes of bipolar disorders. Decisive decreases may occur in the depressive episode of bipolar disorder [46]. Similar to the results obtained for BDNF, we did not observe any difference between the groups with regard to NGF and NT-3 levels. Although Liu et al. reported that NGF level increased in medication-free manic episode patients [13], another study showed that it decreased in the manic episode [7]. It is proposed that NT-3 level increased in different episodes of bipolar disorder [5, 47]. The measurement methods used in these studies are similar to ours. As for the NGF values, a study reported that the standard deviation of NGF values was quite wide while the other study showed that the standard deviation was both quite wide and close to the detection limit as we found in this study. This shows that the method used to measure the NGF level may be an important limitation, which requires the use of more precise methods. On the other hand, the limited number of patients examined in studies conducted to evaluate NT-3 level appears to be a significant limitation. Although it was reported that NGF and NT-3 levels of the peripheral tissue reflected those of the brain [48], these molecules transmit to the blood at low concentrations, which may explain the low levels of these molecules in blood. 

One of the significant findings of our study is the low level of GDNF in bipolar disorder patients. The statistical difference between the patients and the control group persisted even when the confounding factors were taken under control. There are studies in literature which found low levels of GDNF in manic episode patients, as well [49, 50]. We conducted ROC analysis to show that GDNF level might indicate the episode of the disorder and found that area under the the curve was 0.737, which was an interesting finding (Figure 1, Table 3). In the correlation analysis, we did not find any correlation between the GDNF level and the clinical features of the disorder, which was contrary to the findings of some studies [45, 51]. However, serum GDNF level may not reflect the level of GDNF in central nervous system because it can hardly pass the blood-brain barrier. It is known that GDNF production takes place in peripheral tissues [10]. The study of the correlation between the level of GDNF in brain and peripheral tissues requires further attention. Another point is that medication, lithium in particular may have an effect on GDNF levels. It was reported that there was a negative correlation between serum lithium levels and GDNF [40]. In our study, reduced levels of GDNF may be due to lithium used by the patients. 

There were statistical differences between the groups with regard to VEGF, IGF-1 and FGF-2 when confounding factors were not taken under control. However, when the confounding factors were taken under control, the difference between the groups persisted in the IGF-1 level while there were no differences between the groups for VEGF and FGF-2 levels. There are 2 studies reporting that VEGF level increased in manic [16] and depressive [52] episodes of bipolar disorder. The difference between these studies and ours may result from the different confounding factors taken under control. While Lee and Kim [16] reported that statistical difference persisted when BMI was taken under control as the confounding factor, Shibata et al. [52], in another study, did not take BMI and cortisol level under control as confounding factors. Another factor contributing to the difference in the results is that the drugs used may affect the VEGF level. It was reported that lithium reduced the VEGF level [53]. We divided the patient group into 2 subgroups as those who used lithium and who used valproic acid and, found that there was no difference between them with regard to VEGF levels (P = 0.539). Although it was reported in another study [13] that FGF-2 level increased in the manic episode, there was no difference between the groups in our study. The most important difference between the study conducted by Liu et al. [13] and our study is that they did not control the confounding factors. In a postmortem study, it was found that FGF-2 and FGFR1 levels did not change in the brain in bipolar disorder patients [21]. However, further studies are required to reveal the relationship between FGF-2 and bipolar disorder. As for IGF-1 values, Palomino et al. [54] found the same level of IGF-1 in bipolar disorder patients and control group and, Kim et al. [19] found a high level of IGF-1, which is similar to our study. It was reported that the level of IGF-1 produced in the peripheral tissue affected the cognitive functions of the brain [17]. The effect of IGF-1 on the brain is principally achieved via insuline-like growth factor binding protein 2 (IGFBP2) and it was found that the level of this protein was low in the prefrontal cortex of bipolar patients [18]. In addition, the polymorphism occuring in IGF-1 gene may have an important role in the emergence of bipolar disorder [55]. IGF-1 level, which loses its ability to affect the central nervous system as a result of genetical polymorphism and decrease in IGFBP2 level, may increase in order to compensate this. However, the drugs used, particularly lithium, may increase the IGF-1 level [56]. In the light of this information, we found that IGF-1 levels of the patients who used lithium were not different from those of the patients who used valproic acid (P = 0.604). In addition, the ROC analysis we conducted suggested that high level of IGF-1 may be used as a status marker for bipolarmanic episode patients (Figure 2) 

We did not find any literature data examining the level of serum Nrn 1. However, in a recent study, it was proposed that single nucleotid polymorphism of Nrn 1 gene in schizophrenia and bipolar patients was a risk factor for the emergence of these diseases [57]. It was reported that chronic stress reduced the production of neuritin in hippocampus and that the administration of neuritin increased the production of hippocampaldendiritic complex [25]. Nrn 1 and NGF have synergistic effects [58] and elevated level of it after neuronal degradation may show its role on regeneration process [59]. In our study, we found that Nrn 1 level was low in bipolar manic episode patients. In addition, there was a correlation between Nrn 1 and BDNF levels in the control group but there was no correlation between these values in the patient group. The low level of Nrn 1 may lead to a weakness in the repair of damaged neurons or may reflect the neurodevelopmental aspect of bipolar disorder. Hovewer this hypothesis needs to be supported with new research. 

In this study, there are several limitations. One of these was that biochemical parameters are measured in serum and how the serum neurotrophin values reflect the state of the central nervous system is not clear. Second limitation was that the patient group was not drug naive or drug-free and the therapeutic drugs may affect the neurotrophins levels. Third limitation was that we investigated the patients only during the manic episode. Euthymic and depressive episodes could not be evaluated. Fourth limitation was that the study had a relatively small number of participants. 

In conclusion, we found low GDNF and Nrn 1 levels and, high IGF-1 level in bipolar patients with mania. Hovewer, GDNF and IGF-1 may be used as state markers and these findings will be helpful for the development of new treatment strategies which include neurotrophins for bipolar disorder in the future. But these findings must be supported by new reseach involving euthymic episode.

## Acknowledgment/Disclaimer

This study was supported by The Scientific and Technological Research Council of Turkey (TÜBİTAK, Project number: 114S990).

## Conflict of interest 

The authors report no conflicts of interest.
